# Metastatic Renal Cell Carcinoma Change Vascularity

**DOI:** 10.1155/2012/654617

**Published:** 2012-08-30

**Authors:** Takeshi Azuma, Yukihide Matayoshi, Yohsuke Sato, Yujiro Sato, Yasushi Nagase

**Affiliations:** Department of Urology, Tokyo Metropolitan Tama Medical Center, 2-8-29 Musashidai, Fuchu, Tokyo 183-0042, Japan

## Abstract

Several molecular targeted agents have been approved for clinical use for metastatic renal cell carcinoma (mRCC). A case of a 32-year-old woman with mRCC is presented. These tumors could change vascularity by administration of molecular agents. We could select a drug timely based on findings of computed tomography. To our knowledge, this is the first report that tumor's character change induced by molecular targeted agents can be detected and the efficacy of molecular targeted agents can be predicted.

## 1. Introduction

Recently new molecular targeted agents were approved for metastatic renal cell carcinoma (mRCC) [1]. These agents are largely divided into two types. One is multitargeted tyrosine kinase inhibitor (TKI) that inhibits vascular endothelial growth factor receptor [2]. Another is an inhibitor of the mammalian target of rapamycin (mTOR) [3]. However, optimum use of these agents has not been defined for the maximum benefit yet.

We report a case of mRCC, who received sequential therapy of sunitinib and temsirolimus as well as rechallenge of these drugs. We discuss the efficacy and the vascularity change of tumors. 

## 2. Case Report

**Figure 1:**
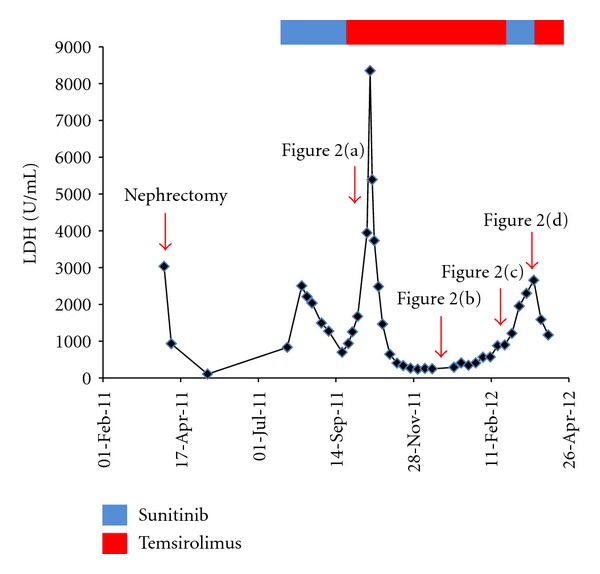
A chart of serum LDH level.

**Figure 2:**
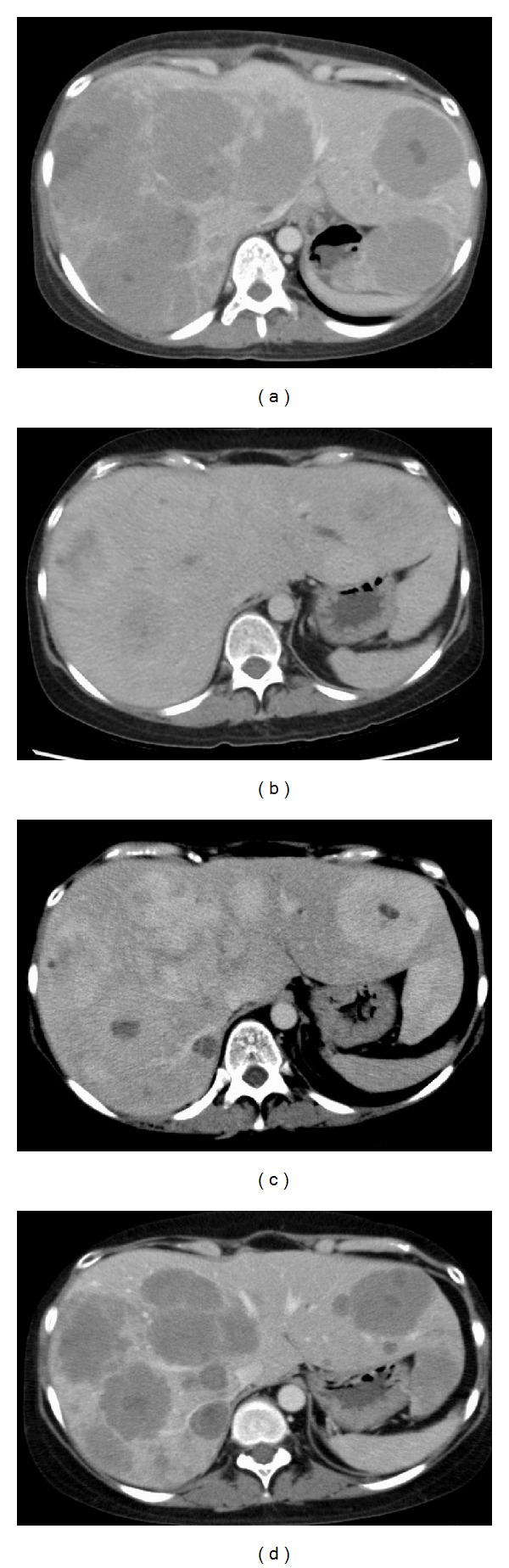
(a) Computed tomography (CT) before first temsirolimus administration. There are multiple metastatic tumors in liver. All tumors are enhanced weakly (64 Hounsfield units). (b) CT 2 months after temsirolimus administration. Most tumors shrink. (c) CT 5 months after temsirolimus administration. Metastatic tumors grow again. All tumors are enhanced strongly (137 Hounsfield units). (d) CT 3 weeks after sunitinib readministration. All tumors turned to be hypovascular again (67 Hounsfield units).

A 32-year-old woman presented with acute abdominal pain and fever. She had undergone a right radical nephrectomy for pT2N0M0, Fuhrman's grade 2, clear cell renal cell carcinoma 4 months ago. Enhanced abdominal computed tomography (CT) revealed multiple hypovascular tumors in the liver. Laboratory findings were increased white blood cells count, as well as elevations of serum C-reactive protein and lactic dehydrogenase (LDH), which was consisted of subtype 1 and 2 (Figure 1). Because these laboratory data were similar to those at the diagnosis of primary RCC, we diagnosed multiple liver metastases of RCC. We started 50 mg sunitinib per day for 4 weeks followed by a 2-week rest period as a first line. Sunitinib is an oral TKI. Serum LDH level was 2500 U/L before sunitinib treatment, then transiently decreased to 694. However, it increased up to 8350 U/L again and CT confirmed progression after 3 months (Figure 2(a)). Sunitinib was stopped, and temsirolimus was administered at a dose of 25 mg per week as a second line. Temsirolimus is an inhibitor of mTOR. Two months after temsirolimus administration, serum LDH level decreased to 233 U/L and CT showed the shrinkage of tumor (Figure 2(b)). Five months after temsirolimus administration, serum LDH level increased to 2290 U/L again and strongly enhanced tumor's progression was confirmed (Figure 2(c)). We thought the tumor was switched from hypovascular to hypervascular tumor and rechallenged sunitinib. Three weeks after sunitinib readministration, CT revealed that all tumors turned to be hypovascular tumor again (Figure 2(d)). Several tumors shrunk and some tumors grew. We rechallenged temsirolimus. One month after temsirolimus rechallenge, serum LDH level decreased to 1199 U/L and CT showed shrinkage of tumors.

## 3. Discussion

Treatment of mRCC has changed dramatically over the past several years by using molecular targeted agents such as TKIs and mTOR inhibitors. However, cure is still rare. Sequential use of these targeted agents is standard because of dose limiting toxicities induced by combination therapy [1]. In our case, mTOR inhibitor as second line could induce an almost complete response because serum LDH level decreased to normal. Although LDH is not usually serum tumor marker for RCC, in this case serum LDH level correlates with tumor volume which was predicted by CT (Figure 1).

The efficacy of rechallenge of molecular targeted drugs, which was failed in a previous line, has been reported [4, 5]. Some mechanisms to restore the sensitivity have been speculated. One is that tumor genetic expression is mediated by exposure of targeted agent. Another is that there are two different subpopulations in RCC: one of TKIs sensitive cells, the other of mTOR inhibitor sensitive cells. However, there is no report that change of tumor's character by targeted drug can be detected actually. In our case, metastatic tumors changed the vascularity quickly after TKI or mTOR inhibitor administration and got the resistance against administered one and had sensitivity to another one. We could prolong survival by using two molecular targeted drugs alternatively based on CT findings and serum LDH level. 

In conclusion, our case showed two important points. The first point is that we should take into consideration the rechallenge of molecular target agents as a treatment option. Second, tumor might change character easily by administration of molecular target drugs. If we can detect these changes like this case, it will help greatly with the choice of agent and prolong survival.

## References

[1] Porta C, Tortora G, Linassier C (2012). Maximising the duration of disease control in metastatic renal cell carcinoma with targeted agents: an expert agreement. *Medical Oncology*.

[2] Motzer RJ, Michaelson MD, Redman BG (2006). Activity of SU11248, a multitargeted inhibitor of vascular endothelial growth factor receptor and platelet-derived growth factor receptor, in patients with metastatic renal cell carcinoma. *Journal of Clinical Oncology*.

[3] Atkins MB, Hidalgo M, Stadler WM (2004). Randomized phase II study of multiple dose levels of CCI-779, a novel mammalian target of rapamycin kinase inhibitor, in patients with advanced refractory renal cell carcinoma. *Journal of Clinical Oncology*.

[4] Roca S, Quivy A, Gross-Goupil M, Bernhard JC, De Clermont H, Ravaud A Efficacy of re-challenging metastatic renal cell carcinoma with mtor inhibitors. *Acta Oncologica*.

[5] Zama IN, Hutson TE, Elson P (2010). Sunitinib rechallenge in metastatic renal cell carcinoma patients. *Cancer*.

